# Analysis of dementia strategic plans in US states

**DOI:** 10.1002/alz.71814

**Published:** 2026-10-19

**Authors:** Matthew Nesvet, Shannon Effler, Natalie Sutton, Zachary Hass, Elizabeth Baker, Kathleen T. Unroe

**Affiliations:** ^1^ University of Oxford Oxford United Kingdom; ^2^ Indiana University School of Medicine Indianapolis Indiana USA; ^3^ Office of Aging Services Washtenaw County Ann Arbor Michigan USA; ^4^ Greater Indiana Chapter Alzheimer's Association Indianapolis Indiana USA; ^5^ School of Nursing and School of Industrial Engineering Purdue University West Lafayette Indiana USA

**Keywords:** Alzheimer's disease and related dementias, cognitive impairment, dementia policy, dementia prevention, dementia risk factors, dementia strategic plans, health‐care policy, mental illness, older adults, public health planning, public health strategy, state health policy

## Abstract

State dementia strategic plans provide policy and practice recommendations to reduce the risk, progression, and impact of dementia. Yet there is little systematic understanding of the contents of state plans nor how they aim to influence dementia strategy. This article analyzes state dementia plans, identifying five underspecified dementia policy areas in recent state plans and inconsistency in how states monitor the implementation of plans for effectiveness.

## 1 INTRODUCTION

In the United States, increasing numbers of people live with dementia or care for others with the illness. Researchers and policy advocates have called for local, state, and federal lawmakers to improve care and support for people living with dementia and those who care for them through policy change. To provide lawmakers, leaders, and professionals in different sectors and industries guidance on which policies and practices may be most impactful, federal and state lawmakers have directed the authorship of state‐specific dementia strategic plans to bolster the nation's capacity to improve dementia care and prevention. Since the early years of the twenty first century, every US state and the District of Columbia and Puerto Rico has released at least one dementia strategic plan. Many plans were created after the US Congress passed the Building Our Largest Dementia (BOLD) Infrastructure for Alzheimer's Act (P.L.115‐406), signed into law in 2018, though we identified dementia strategic plans extending several decades before BOLD Act funding was made available to states.^1^ For example, South Carolina released their first dementia plan much earlier, in 1993.

The authors of state dementia plans and national dementia strategies typically carry out yearlong or more strategic planning processes that result in recommendations for dementia policy change. But how plans are made has elicited little scholarly interest compared to the large evidence base of research on best practices in dementia care and support that dementia planners marshal when making policy recommendations. Dementia is an international priority and there are multiple examples of country‐specific dementia plans. Alongside these, a small group of scholars have carried out cross‐national comparisons of countries’ dementia plans.^2^, ^3^, ^4^, ^5^, ^6^ But there are few comparable efforts to gather and analyze the content of US states’ dementia plans or assess plan characteristics such as whether state plans include goals and timelines for recommendations to be implemented or how long state plans endure before they are updated. This analysis obtained and reviewed the 25 most recently developed US state or territory dementia strategic plans to identify current priorities for dementia prevention and care at the state level and review strategies for implementation and monitoring of plan effectiveness.

## 2 METHODS

This paper analyzes recent and historical dementia strategic plans from 52 US state, federal district and territory dementia strategic plans. All plans were selected from states and territories (Puerto Rico and Washington, DC) that produced dementia‐specific strategic plans from 1993, the first year in which we identified a state dementia plan's release, to August 2025. The final plan that we reviewed was released in 2025; we concluded data collection in August 2025.

### 2.1 Search strategy and selection criteria

We gathered and archived the text of state, territory, and federal district dementia strategic plans—any plan that specifically and exclusively devised policy strategies to reduce the incidence and/or manage the symptoms of dementia at the state policy level. We excluded plans developed at the city or county level. In addition to state plans, we included US territory (Puerto Rico and District of Columbia) dementia strategic plans. We excluded plans that did not focus exclusively on Alzheimer's disease and related dementias (ADRD), for instance state aging policy plans that only partially focused on preventing, diagnosing, and treating ADRD among other aging policy concerns.

To identify a comprehensive set of dementia plans for each jurisdiction, we found and reviewed states’ most recent dementia‐specific plans to learn whether the plan was their initial plan or if any earlier plans existed. We also noted when plans included information about the years when earlier plans were released. To identify each state's most recent plan, we searched the internet for references to state, district, and federal territory dementia plans using Google, LexisNexis, and Overton. We then contacted state officiagls at agencies associated with their recent dementia plan or the state's Alzheimer's Association chapter for assistance in gathering historic and recent plans, particularly when the recent plan's text did not include information about earlier plans or the full texts of a jurisdiction's dementia plans could not be found online or could not be accessed due to broken web links. We also reviewed state dementia plan documents that the national Alzheimer's Association shared with us to confirm each of the plans they shared was included in our archive. We then compiled metadata on the plans that we had gathered including release dates of all initial dementia strategic plans, dates of states’ most recent dementia strategic plans, dates of new plans and plan updates published in the interim, and the number of years that had passed from when a state or territory first released a dementia‐specific plan to the year of its most recent plan (Table 1).

**TABLE 1:** Archived dementia plans in US states and territories (*n* = 52).^a^

1 plan (*n* = 18)	AL (2012), AZ (2016), DC (2024), DE (2014), KS (2020), ME (2024), MT (2016), NE (2016), NJ (2016), OH (2024), OR (2012), PA (2014), PR (2015), SD (2018), VT (2022), WA (2023), WV (2011), WY (2018)
2 plans (*n* = 18)	AK (2014, 2021), AR (2011, 2022), CA (2011, 2020), CO (2010, 2022), FL (2013, 2020), GA (2014, 2020), ID (2013, 2023), KY (2008, 2017), LA (2009, 2023), MA (2012, 2019), MD (2012, 2022), MO (2010, 2022), MS (2015, 2020), NC (2016, 2020), ND (2007, 2022), NH (2013, 2015), TN (2009, 2021), TX (2019, 2024),
3 plans (*n* = 10)	CT (2013, 2020, 2023), HI (2013, 2020, 2023), IA (2008, 2011, 2022), MN (2011, 2018, 2022), NM (2013, 2017, 2022), OK (2009, 2016, 2023), RI (2013, 2019, 2024), SC (1993, 2009, 2023), UT (2012, 2018, 2023), WI (2014, 2019, 2024)
4+ plans (*n* = 6)	IL (2014, 2017, 2020, 2023), IN (2013, 2018, 2021, 2022, 2023), MI (2003, 2009, 2019, 2023), NV (2013, 2015, 2017, 2019, 2021, 2023), NY (2009, 2011, 2013, 2015, 2017, 2019, 2021), VA (2011, 2015, 2019, 2024)
Jurisdictions	52
Plans and plan updates released January 2022–August 2025	36
Median (mean) years between plan releases	5 (5.82)
Median (mean) time from first plan release to most recent plan release	10 (10.62)

^a^ Recent and initial state and territory dementia strategic plans that we gathered are archived and available online in the Oxford University Research Archive (ORA), https://ora.ox.ac.uk.

### 2.2 Evaluation of plans and recommendations

We created an archive of the full text of all state and territory dementia strategic plans that we gathered (available upon request). We then created a dataset with information on the number of plans and plan updates that each state or territory released from initial to most recent plan and their years of publication. We then undertook a first read of all initial and most recent plans and met with two groups to discuss their content. A set of policy domains felt to be most relevant to advancing dementia policy was developed and reviewed through conversations with the steering committee for the state of Indiana dementia strategic plan and experienced state dementia strategic plan authors and policy professionals (*n* = 6) in a focus group meeting organized by the Alzheimer's Association national office. In the Alzheimer's Association focus group, we also discussed key elements and recommendations found in historical and recent dementia plans, learning from association staff and leadership about how states’ plans have changed over time. The domains that we identified from these conversations to be of interest to stakeholders included nine categories, such as quality of dementia care, home and community‐based support, workforce and training, and strategies for disseminating information and recommendations that plans contain, for example, deploying public dashboards (Table 2).

**TABLE 2:** Research framework for content analysis.

Recommended policy domain	We categorized recommendations that we found in state plans by policy domain, coding each recommendation as one of the following 9 types: Quality of care Workforce and training Behavioral health Home and community‐based support Reimbursement policy Awareness Risk reduction Data sources Implementation
Actionable recommendation	We assessed each policy recommendation for its specificity, categorizing some as actionable, others as general, only. For example, the Washington, DC, plan urged “use [of] evidence‐based care for a person living with dementia,” which we categorized as a general recommendation lacking an action step, whereas Texas’ planners wrote, “implement Alzheimer's Association Dementia Care Practice Recommendations,” an action step that refers to a set of specific written guidances.
Release date for each plan	Initial, most recent
Number of released plans	New plans and plan updates were counted

For analysis, we selected all dementia plans released in the prior 4 years, 2022 to 2025, in all states except Indiana^7^ (*n* = 25). Indiana was excluded as our study was undertaken to inform the development of the Indiana state plan. We focused on recent plans (2022–2025) to ensure that our findings reflect the current state of dementia plan priorities.

We analyzed recent plans’ content to identify policy recommendations that the plans contained, organizing their recommendations by policy type. To make our study most relevant to Indiana and other states’ dementia policy planners, we conducted a deductive thematic analysis using the identified policy domains.

When reviewing plans, two authors (M.N. and E.B.) used a consensus approach to identify which plans made recommendations within each policy domain. We then used a consensus approach to differentiate when plans only called for a problem to be addressed and when a particular solution was recommended to address that problem.

## 3 RESULTS

Our findings reporting which dementia strategic plans address each policy domain either with a specific recommendation for dementia policy change or calling for a problem to be addressed without recommending a specific policy to address it are summarized in Tables 3 and 4. Table 3 provides information about the number of state dementia plans that address each of nine policy domains and lists the most frequently recommended action in each domain. Table 4 summarizes the share of dementia plans that provide specific, actionable recommendations for the nine policy domains and which plans either address these domains in general terms only or not at all. Fewer than half of the dementia plans updated in the previous 4 years recommended either specific policy changes or urged policy attention without suggesting specific actions to reduce dementia risk factors (risk reduction), effectively implement and monitor the impact of the dementia plan (implementation strategy), or address serious behavioral health symptoms or comorbidities among people living with dementia (behavioral health).

**TABLE 3:** Policy recommendations by type, frequency, and most common recommendation.

Recommendation type	Frequency	Most common recommendations (3+ plans include)
Quality of care	25	Expand or audit telehealth programs in rural areas, where provider shortages limit older adults’ access to dementia care (AR, ID, SC, VA)
Workforce and training	25	Train medical providers and care workers to deliver culturally appropriate care through specific program recommendations (AR, CO, ID, MD, ME, MO, SC, UT, VT, WA) and initiate or expand dementia‐specific training for direct service workers (including home health aides) and community health workers (AR, DC, HI, ID, MD, ME, MO, NV, TX)
Home and community‐based support	25	Implement respite programs to support caregivers and/or implement a respite care grant program (AR, ID, MO, NM)
Awareness	25	Implement culturally specific and/or culturally relevant dementia awareness campaigns to increase treatment access and/or awareness of risk factors (CO, AR, ID, IL, MN, RI, WA) and make dashboard with resources, statistics, and metrics (CO, CT, DC, HI, IA)
Reimbursement	17	Incentivize detection and quality of care with increased reimbursement rates if care benchmarks met (DC, MO, NV)
Original data sources	17	Implement or monitor Behavioral Risk Factor Surveillance System surveys of people living with dementia and/or caregivers (AR, DC, HI, IL, LA, ND, NV, TX, UT, VA, VT, WA, WI)
Risk reduction	10	Address modifiable risk factors related to lifestyle and/or head injuries (DC, AR, LA)
Implementation strategy	9	Create a (new only) director‐level state dementia coordinator position to coordinate dementia plan implementation and strategy (AR, MD, RI)
Behavioral health	7	Not applicable (no common behavioral health recommendations were identified in 3 or more plans)

**TABLE 4:** Policy recommendations specific versus actionable.

Recommendation type	Frequency (count)	Specific action steps suggested	Made general only recommendation(s)	Did not address
Quality of care	25	52%	48%	0%
Workforce and training	25	100%	0%	0%
Home and community‐based support	25	56%	44%	0%
Awareness	25	68%	32%	0%
Reimbursement	17	40%	28%	32%
Original data sources	17	60% collected original data, then included analysis of the collected data in their plans		40% did not report collecting any original data
Dementia risk reduction	10	40%	0%	60%
Implementation strategy	9	64%	0%	36%
Behavioral health	7	20%	8%	72%

State and territory dementia strategic plans universally recommended policy changes to improve quality of care for people living with dementia (100%); recruit, retain, and effectively train states’ dementia workforces (100%); improve home and community‐based support for people living with dementia (100%); and increase public and professional awareness of dementia (100%; Table 3). Fewer plans recommended policy changes related to reimbursement (68%), collected original data to support policy planning or recommended new data collection and analysis (68%), recommended policy changes to reduce risk factors associated with onset or severity of dementia symptoms (40%), recommended ways to implement and monitor how the plan's dementia strategy is disseminated (36%), or recommended policies related to behavioral health and serious behavioral health symptoms among people living with dementia (28%).

Plans that recommended dementia policy change varied in whether their suggestions contained prescriptive policy advice or general calls for action to be taken. For example, plans that recommended state lawmakers fund state dementia coordinator positions provided legislatures with actionable policy recommendations. By contrast, some plans articulated general calls for change. For example, some plans called for increasing public awareness of dementia risk factors without suggesting specific steps that legislatures should take to achieve this, such as funding public awareness campaigns.

We found that plan authors more frequently recommended specific actions be taken in some policy domains, such as dementia workforce and training, whereas in other domains, such as dementia risk reduction, general recommendations without specific action steps were more the norm (Table 4). All reviewed plans provided specific, actionable recommendations for dementia workforce training (100%). Fewer plans made specific, actionable recommendations to improve dementia awareness (68%); undertook original data collection for dementia policy planning purposes (68%); or made specific, actionable recommendations to improve quality of dementia care (52%), home and community‐based supports for people living with dementia (56%), policies to reduce dementia risk factors (40%), how state dementia strategies can be implemented or monitor implementation (36%), or how to better address behavioral health challenges and services for people living with dementia (20%).

By contrast, 48% of dementia strategic plans made general calls to alter state dementia policy to improve quality of care without offering specific, actionable recommendations; likewise, 44% of plans made general recommendations about improving home and community‐based support for people living with dementia; and 32% of plans offered general recommendations to increase dementia awareness.

Some aspects of dementia policy and planning went unmentioned in a majority of state plans. We found that 64% of plans provided no recommendations—actionable or general—about how to effectively implement the state's dementia strategic plan and monitor its implementation for effectiveness. We also found that 60% of the 25 dementia strategic plans neither suggested addressing modifiable risk factors for dementia nor provided any actionable recommendations about how to reduce risk.

## 4 DISCUSSION

Most states have committed resources to develop state dementia strategic plans to draw attention to the policy importance and impact of dementia and provide guidance for policy to address dementia. This analysis of 25 recently developed or revised dementia strategic plans reveals multiple common themes. All plans emphasize the need to enhance the dementia care workforce and include actionable policy recommendations in this domain. Other policy domains receive less attention, or are described in plans but actionable policy recommendations are not included. This may reflect current priorities of advocates and policymakers, including economic considerations, or a lack of evidence‐based policies that plan authors could identify and promote. Further, authorship teams for dementia strategic plans are varied and inclusion of different expertise and perspectives could broaden or deepen the focus of policy recommendations. Several policy implications of this dementia strategic plan review are discussed below.

Policy domains that many plans leave unaddressed or only generally call on policymakers to implement change may lack specific, evidence‐based solutions and require more research to identify effective responses. For example, few plans recommended addressing behavioral health for people living with dementia. This may be a sign that more research is needed to identify policies and practices that can be implemented at the state level to improve diagnosis, treatment, and care for people living with ADRD and co‐occurring serious mental illnesses and other behavioral health challenges. ^8^

Another factor that may influence under‐development of certain domains may be due to the economic or political costs of implementing effective change in these areas. For example, there is a body of research on the impact of factors such as nursing home ownership and nursing home staffing including reducing staff turnover.^9^, ^10^, ^11^, ^12^ These may be areas that feel too difficult to address at a state level or which have significant political or economic considerations. Likewise, plans that address ways to reduce dementia risk may focus on modifiable lifestyle factors such as diet and exercise more than policy areas such as environmental planning and regulation that could reduce exposure to particulate air pollution, again due to challenges related to costs and political interests.

Plan authors may also lack expertise in some areas of potential dementia policymaking, which could cause some plans to focus on domains that planners possess the most expertise in, such as health care and human services. Expansion of authorship teams and advisory groups, both from within state government and outside of it, for developing dementia strategic plans that include expertise in some of these other domains may change the content of recommendations.

We found that many plans do not contain evidence‐based strategies to monitor or evaluate plans’ implementation, or timed benchmarks to measure and report successful implementation. This accords with a general lack of available research on state dementia policy planning to support state dementia plan makers, which this article aims to help address. State planners can consult existing state plans that recommend monitoring and evaluating state progress in implementing dementia policy and improving capacity and care. For example, the Hawaii 2035: State Strategic Plan on Alzheimer's Disease and Related Dementias recommends tracking state progress by creating a regularly updated, publicly available Healthy Brain Tracker, which was released online in May 2025.^13^ Policymakers can also look to plans that include dissemination strategies to increase their effectiveness, such as publicly sharing plan findings and recommendations in stakeholder forums or informing legislatures about progress and needs through issuing regular reports and plan updates. Planners may also examine whether research on dementia plans and implementation strategies from other countries can also inform US states’ planning efforts.^5^, ^14^ Some state plans and updates also include information on earlier plan goals and report progress achieving these goals or whether plan recommendations were effectively implemented. For example, after the first Washington State Plan to Address Alzheimer's Disease and Other Dementias was released in 2016, plan authors created a workgroup to implement the plan's recommendations. The subsequent state plan (2023‐2028) provides information about which recommendations from the earlier plan were implemented and links to a workgroup website that tracks implementation progress for each recommendation made in the earlier and current state plans. At the same time, much remains to be learned about which practices engaging with policymakers and other stakeholders may be most effective in implementing calls for policy and practice change.

Given that US BOLD Act funding and other mechanisms have made state and federal resources available for dementia policy planning and states and stakeholders have expended large amounts of time and effort creating dementia strategic plans, monitoring the plans’ dissemination and implementation may enable later modifications to plan recommendations and dissemination practices to address shortcomings. For example, in several states that we reviewed, the dementia strategic plan itself was unavailable online for policymakers and other stakeholders to access due to broken web links. In some state dementia plans, more clearly defined mechanisms for both plan implementation and monitoring which recommendations were successfully implemented and which remain incomplete could lead to a better understanding of the positive outcomes and encountered challenges of implementing each plan.

Further, we found risk reduction for developing dementia was only addressed in 40% of state plans, and that the remaining plans did not include even general recommendations to tailor state policies to reduce dementia risk among people not yet diagnosed with ADRD. Even among the 40% of plans that did address risk reduction with specific actions, few plans were found to include policy recommendations beyond raising awareness of modifiable lifestyle factors that reduce risk for dementia such as weight reduction or tobacco cessation.^15^ No plans comprehensively addressed the 12 factors that researchers have shown contribute to dementia risk, nor did any plan identify possible emerging risk factors or provide lawmakers with any policy options to address these.^15^ In the last few years, new risk factors have been identified that state policy can address, for example, most recently, new evidence that particulate air pollution and non‐adherence to recommended shingles vaccine schedules increases dementia risk in older adults.^16^ Plan authors can expand the scope and potentially effectiveness of state strategies to reduce dementia risk by providing more comprehensive information and policy guidance about dementia risk factors. Planners may also consider whether it is appropriate to link dementia plans to other state plans that may address dementia risk factors, for example, recommendations to better fund tobacco control and cessation support or to improve pollution control, zoning laws that limit housing close to highways, and environmental remediation policy and planning.

We also found fewer than half (40%) of state plans addressed reimbursement rates for dementia services with specific recommendations for policy interventions to increase rates when needed, though providers and policymakers very frequently refer to these reimbursement rates to explain lack of availability of services for persons living with dementia.^17^

In addition, we found that both quality of care and home and community‐based support were frequently mentioned in state strategic plans, but that slightly fewer than half of the reviewed plans contained specific action steps for policymakers to improve quality of care or home and community‐based support for persons living with dementia. This was particularly noteworthy given that all plans did include language referencing the importance of state policy encouraging improvements in these domains.

Finally, we found that the nexus of dementia and mental and behavioral health was absent in state plans, including services tailored to persons living with dementia and intellectual disabilities, the diagnosis and management of serious mental illness among persons living with dementia, and other opportunities to place state mental health resources, policies, and plans into dementia policy. Such dual diagnosis populations may encounter special challenges to accessing effective dementia care that state policy could help address. Examples include requiring or making available additional training for medical providers and caregivers; outreach to organizations that serve persons living with mental, behavioral, and intellectual disabilities; and incentivizing research and program development for dual diagnosis populations.

This review of recent dementia strategic plans was intended to report on current policy priorities identified at the state level and to guide development for plan authors, specifically the Indiana state plan. One limitation to this study is that the use of deductive analysis to assess plans’ content means that additional policy themes may have gone unreported. An additional limitation is that this analysis only covers recent plans’ content, whereas a historical analysis may have revealed changes over time to plans’ thematic contents that could be informative to policymakers.

## 5 CONCLUSION

This analysis of 25 recently developed or revised dementia strategic plans reflects many of the current policy priorities to support people living with dementia and their care partners. While there are some important, nearly universal themes addressed in every recent plan such as expansion of home and community‐based services and improvements to quality of dementia care, there is often a lack of specific policy recommendations. Other areas, such as behavioral health and dementia risk factor reduction, are often unaddressed. Continuing to diversify the government and community stakeholder involvement in the development of plans, and the addition of clear milestones and implementation plans, may enhance the ability of these plans to guide policy reform to address the needs of this population.

## FUNDING INFORMATION

The authors received no financial support or funding for the research, authorship, and/or publication of this manuscript.

## CONFLICT OF INTEREST STATEMENT

Matthew Nesvet has no conflicts to report. Shannon Effler has no conflicts to report. Natalie Sutton has no conflicts to report. Zachary Hass has no conflicts to report. Elizabeth Baker has no conflicts to report. Dr. Kathleen T. Unroe is a founder and CEO of Probari, a healthcare startup designed to improve clinical care services in nursing homes. Author disclosures are available in the Supporting Information.

## Supporting information



Supporting Information
